# Concurrent Genetic Alterations and Other Biomarkers Predict Treatment Efficacy of EGFR-TKIs in EGFR-Mutant Non-Small Cell Lung Cancer: A Review

**DOI:** 10.3389/fonc.2020.610923

**Published:** 2020-12-10

**Authors:** Yijia Guo, Jun Song, Yanru Wang, Letian Huang, Li Sun, Jianzhu Zhao, Shuling Zhang, Wei Jing, Jietao Ma, Chengbo Han

**Affiliations:** Department of Clinical Oncology, Shengjing Hospital of China Medical University, Shenyang, China

**Keywords:** non-small cell lung cancer, concurrent genetic alteration, epidermal growth factor receptor tyrosine kinase inhibitors, PD-L1 expression, BIM polymorphism

## Abstract

Epidermal growth factor receptor tyrosine kinase inhibitors (EGFR-TKIs) greatly improve the survival and quality of life of non-small cell lung cancer (NSCLC) patients with EGFR mutations. However, many patients exhibit *de novo* or primary/early resistance. In addition, patients who initially respond to EGFR-TKIs exhibit marked diversity in clinical outcomes. With the development of comprehensive genomic profiling, various mutations and concurrent (*i.e.*, coexisting) genetic alterations have been discovered. Many studies have revealed that concurrent genetic alterations play an important role in the response and resistance of EGFR-mutant NSCLC to EGFR-TKIs. To optimize clinical outcomes, a better understanding of specific concurrent gene alterations and their impact on EGFR-TKI treatment efficacy is necessary. Further exploration of other biomarkers that can predict EGFR-TKI efficacy will help clinicians identify patients who may not respond to TKIs and allow them to choose appropriate treatment strategies. Here, we review the literature on specific gene alterations that coexist with EGFR mutations, including common alterations (intra-EGFR [on target] co-mutation, TP53, PIK3CA, and PTEN) and driver gene alterations (ALK, KRAS, ROS1, and MET). We also summarize data for other biomarkers (*e.g.*, PD-L1 expression and BIM polymorphisms) associated with EGFR-TKI efficacy.

## Introduction

Lung cancer is the most prevalent cancer type and is one of the leading causes of cancer-related death (1). The discovery of epidermal growth factor receptor tyrosine kinase inhibitors (EGFR-TKIs) and the fact that most patients with EGFR-mutant non-small cell lung cancer (NSCLC) can benefit from TKI treatment have dramatically changed the therapeutic approach for NSCLC. EGFR mutations occur in approximately 10–35% of lung adenocarcinomas (2), with a higher prevalence of about 40–55% in East Asian patients (3). Because EGFR-TKIs, including first-generation gefitinib, erlotinib, and icotinib, second-generation afatinib and dacomitinib, and third-generation osimertinib, have demonstrated higher objective response rates (ORR) and prolonged progression-free survival (PFS) compared to standard chemotherapy (4–7), they have become the first choice for patients with advanced EGFR-mutated NSCLC. However, approximately 20–30% of patients exhibit primary resistance to EGFR-TKIs (4). Furthermore, even in patients with an initial response, significant heterogeneous outcomes have been observed. Some patients only respond for a few weeks, while others may benefit for years without progression.

Comprehensive genomic profiling has allowed us to understand various mutations and co-occurrence of genomic alterations in NSCLC and explore their impact on clinical outcomes. Many studies have demonstrated that concurrent genetic alterations potentially impair TKI efficacy and partly explain the heterogeneous patient outcomes (8–10). Hong et al. (8) analyzed 58 EGFR-mutant patients with metastatic NSCLC treated with first-line EGFR-TKIs. They demonstrated that concomitant mutations are widespread and significantly associated with reduced ORR and shorter overall survival (OS). Sato et al. (11) found that PFS for EGFR-TKIs in EGFR-mutant lung adenocarcinoma was associated with the number of concurrent genomic alterations. Significantly poorer PFS was observed in patients with four or more genomic alterations. The types of genomic alterations coexisting with EGFR mutations may also be associated with EGFR-TKI efficacy. For instance, Wang et al. (12) showed that patients with mutations in EGFR and oncogenes have shorter PFS compared to those with mutations in EGFR and tumor suppressor genes and patients with EGFR mutations only (4.7 *vs*. 9.3 *vs*. 13.2 months), which is consistent with other reports (13–15). In addition, EGFR mutations are thought to be mutually exclusive with other oncogenic drivers. However, recent studies have demonstrated that although at a small percentage (16–18), additional driver alterations coexist with EGFR mutations in TKI therapy-naïve NSCLC and may impact EGFR-TKI efficacy and partly explain the intrinsic resistance in some patients (19).

**Figure 1:**
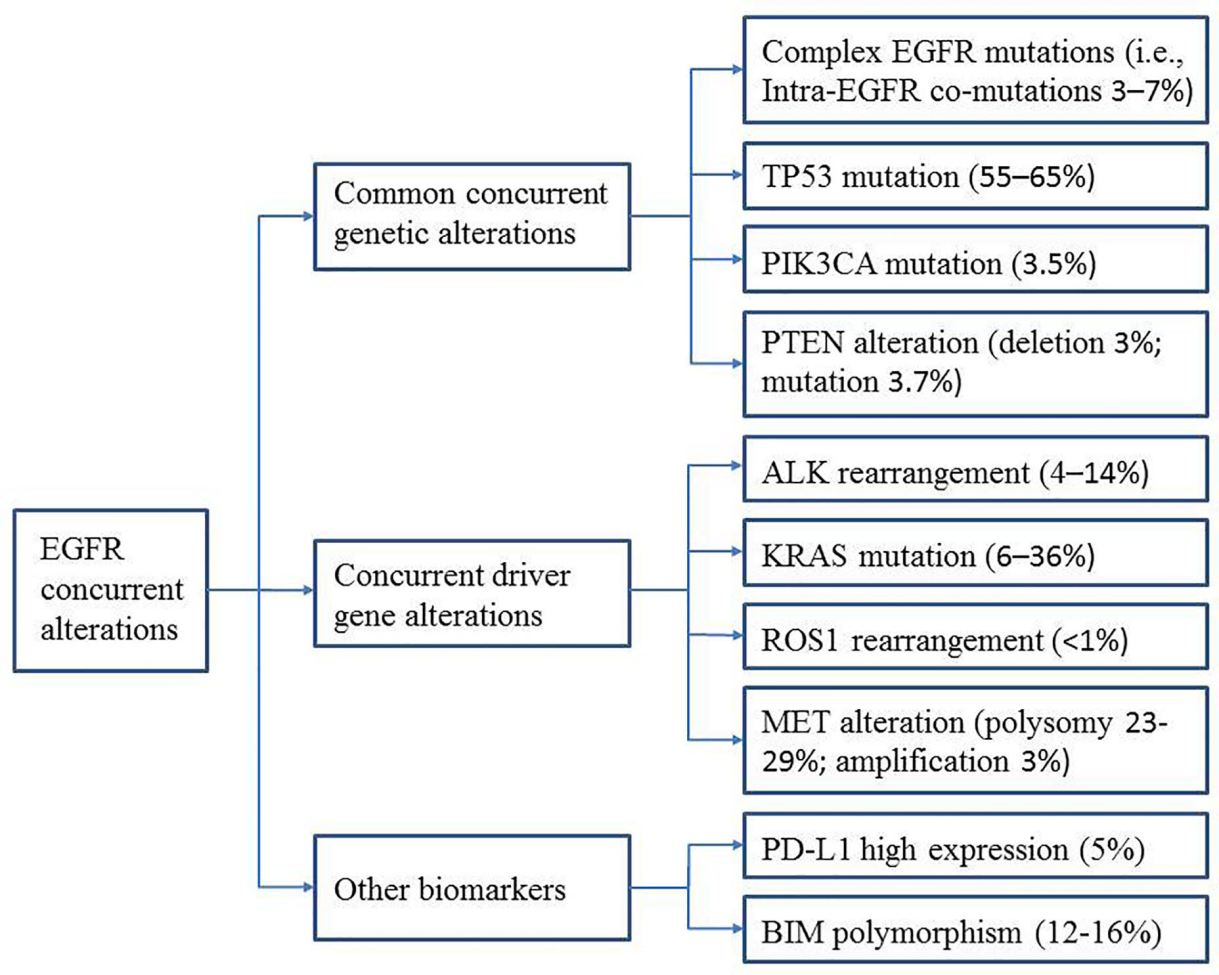
Overview of EGFR concurrent alterations.

A greater understanding of the relationship between specific concurrent genes and TKI efficacy is urgently needed and will help predict clinical outcomes and guide clinicians in selecting the best treatment strategies for patients with these concurrent mutations. In this review, we describe the recent progress and future perspectives in this area, focusing on common concurrent genetic alterations [intra-EGFR (on target) co-mutation, TP53, PIK3CA, and PTEN] and concurrent driver gene alterations (ALK, KRAS, ROS1, and MET). Furthermore, we summarize the data on other biomarkers (*e.g.*, PD-L1 expression and BIM polymorphisms) that appear to be associated with EGFR-TKI efficacy (**Figure 1**). The main characteristics of the studies described are summarized in **Tables 1** and **2**.

**Table 1:** Tyrosine kinase inhibitor responses of patients with concurrent alterations from literature review.

Case	Source	Gender	Age	Smoking history	Histology	Stage	Genotype	Targeted agents (Treatment line)	Response	PFS, mo	Year
1	Benesova (20)	Male	74	Yes	Squ.	IIIA	EGFR 19del+KRAS	Gefitinib	PR	3	2010
2	Benesova (20)	Male	65	No	Ade.	IV	EGFR 19del+KRAS	Gefitinib	PR	7	2010
3	Benesova (20)	Female	71	No	Ade.	IV	EGFR L858R+KRAS	Erlotinib	PR	5	2010
4	Zhuang (21)	Male	53	No	NSCLC	IV	EGFR L858R+KRAS+ROS1	Crizotinib (Second)	PD		2019
4	Zhuang (21)	Male	53	No	NSCLC	IV	EGFR L858R+KRAS+ROS1	Icotinib (Third)	PR	27.5	2019
5	Zhuang (21)	Male	52	Yes	NSCLC	IV	EGFR L858R+KRAS+ROS1	Gefitinib (First)	PR	12.7	2019
6	Lai (22)		62	No	NSCLC		EGFR L858R+MET amplification	Erlotinib (First)	PD	<1	2019
6	Lai (22)		62	No	NSCLC		EGFR L858R+MET amplification	Crizotinib (Second)	PR		2019
7	Li (23)		68	Yes	Squ.	IV	EGFR 19del+MET amplification	Icotinib	PD		2019
7	Li (23)		68	Yes	Squ.	IV	EGFR 19del+MET amplification	Crizotinib	PR		2019
8	Gainor (24)		73	No	Ade.	IV	EGFR L858R+MET amplification	Erlotinib	PD	<1	2016
8	Gainor (24)		73	No	Ade.	IV	EGFR L858R+MET amplification	Erlotinib plus crizotinib	PR		2016

PFS, progression-free survival; mo, months; Squ, Squamous cell carcinoma; PR, partial response; Ade, adenocarcinoma; NSCLC, non-small cell lung cancer; PD, progressive disease.

**Table 2:** Main characteristics of available studies.

Source	Year	Targeted agents (Treatment line)	Patients	Group or subgroup		N		ORR		DCR	Median PFS, mo	Median OS, mo	Rebiopsy with EGFR T790M
Complex EGFR mutations (intra-EGFR co-mutation)													
Zhang (25)	2018	EGFR-TKIs (First)	EGFRm advanced lung ade.	Intra-EGFR mutations		46		52.2%		71.7%	9.5		
				Group A (19del+L858R)		12		75%		100%	18.2		
				Group B (19del/L858R+atypical mutations)		15		60%		86.7%	9.7		
				Group C (double atypical mutations)		7		71%		85.7%	9.6		
				Group D (with a primary drug-resistant pattern)		12		8.3%		16.7%	1.4		
Keam (26)	2013	Gefitinib/erlotinib	EGFRm locally advanced or metastatic NSCLC	Intra-EGFR with classical mutation		16		68.8%		93.8%	8.1		
				Single classical mutations (19del/L858R )		269		74.8%		89.3%	11.9		
Wu (27)	2008	Gefitinib (≥First)	EGFRm stage IIIB/IV lung ade.	Intra-EGFR co-mutations		19		63%			8.1	22.4	
				Co-mutations with classical mutation (19del/L858R )		12		83%			12.7	24.7	
				Co-mutations without classical mutation		7		29%			4.9	12.3	
				Single classical mutations (19del/L858R )		168		73%			8.1	16.4	
TP53													
Kim (28)	2018	Gefitinib/erlotinib/ afatinib	EGFRm advanced NSCLC	Concurrent TP53 mutations		43					12.5		
				TP53 wild type		32					14.7		
Canale (29)	2017	Gefitinib/erlotinib/ afatinib/dacomitinib (First)	EGFRm NSCLC	Concurrent TP53 mutations		37				70%			
				TP53 wild type		86				88%			
				Concurrent TP53 exon 8 mutations						42%	4.2	16.2	
				TP53 exon 8 wild type						87%	12.5	32.3	
				19del with TP53 exon 8 mutations							4.2	7.6	
				19del with TP53 exon 8 wild type							16.8	NR	
VanderLaan (30)	2017	Gefitinib/erlotinib/ afatinib	EGFRm stage IV recurrent NSCLC	Concurrent TP53 mutations		7		71.4%			7	25	1/4 (25%)
				TP53 wild type		9		88.8%			15	32	3/5 (60% )
				19del with TP53 mutations		4		75%			6.5	15.5	1/4 (25%)
				19del with TP53 wild type		5		100%			19	NR	2/2 (100% )
Labbé (31)	2017	Gefitinib/erlotinib (First)	EGFRm advanced or recurrent NSCLC	Concurrent TP53 mutations		24		54%		96%	10		5/11(45%)
				TP53 wild type		36		67%		85%	16.8		9/13 (69% )
Labbé (31)	2017	Third generation EGFR-TKIs	Acquired T790M	Concurrent TP53 mutations		3		100%		100%			
				TP53 wild type		8		88%		88%			
Kim (28)	2018	Third generation EGFR-TKIs	Acquired T790M	Concurrent TP53 mutations		50					8.9	17.8	
				TP53 wild type		32					12.8	26.6	
PIK3CA													
Eng (32)	2015	EGFR-TKIs	EGFRm ade.	Concurrent PIK3CA mutations		10		83% (5/6)			7.8	14.6	
				PIK3CA wild type		32		62% (18/29)			11.1	14.5	
Wu (33)	2016	Gefitinib/erlotinib/ afatinib	EGFRm ade.	Concurrent PIK3CA mutations		6		66.7%		83.3%	12	25.1	
				PIK3CA wild type		338		78.7%		84%	8.8	21.4	
PTEN													
Kim (28)	2018	Third generation EGFR-TKIs	Advanced NSCLC with acquired T790M		Concurrent PTEN mutations		3				2.6	13.2	
				PTEN wild type		79					10.3	24.3	
ALK													
Yang (34)	2014	EGFR-TKIs (First)	Advanced NSCLC	Concurrent EGFR and ALK alterations		13		80%			11.2		
Lou (35)	2016	EGFR-TKIs (First)	EGFRm advanced NSCLC	Concurrent EGFR and ALK alterations		10		80%			11.2	18.5	
				Single EGFR mutation		84		65.5%			13.2	21.3	
		ALK-TKIs (≥Second)	Advanced NSCLC with ALK rearrangement	Concurrent EGFR and ALK alterations		5		40%			1.9		
				Single ALK rearrangement		23		73.9%			6.9	23.7	
Zhao (36)	2019	First generation EGFR-TKIs	EGFRm advanced NSCLC	Concurrent EGFR and ALK alterations		19		63.2%			10.3	36.2	
				Single EGFR mutation		95		62.1%			11.4	27.1	
		Crizotinib	Advanced NSCLC with ALK rearrangement	Concurrent EGFR and ALK alterations		12		66.7%			11.1		
				Single ALK rearrangement		60		65%			12.5	36.2	
KRAS													
Zhuang (21)	2018	EGFR-TKIs	EGFRm NSCLC	Concurrent EGFR and KRAS alterations		8		62.5%			8.2		
				Single EGFR mutation		100					9.6		
Rachiglio (37)	2019	EGFR-TKIs	Advanced or metastatic NSCLC	concurrent EGFR and KRAS alterations		14							
				VAF of KRAS higher than EGFR		6		16.7%			2.42		
				VAF of KRAS lower than EGFR		8		57.1%			11.09		
MET													
Noro (38)	2015	Gefitinib	EGFRm ade.	MET FISH positive		11					7.6	16.8	
				MET FISH negative		24					15.9	33	
Lai (22)	2019	EGFR-TKIs (First)	EGFRm NSCLC	MET FISH high		39		74.4%			12.2		
				MET FISH low		115		53.9%			13.1		
PD-L1													
Su (39)	2018	EGFR-TKIs	EGFRm advanced NSCLC	PD-L1 strong expression				35.7% (5/14)			3.8		
				PD-L1 weak expression				66.7% (12/18)			6		
				PD-L1 negative expression				67.3% (35/52)			9.5		
Hsu (40)	2019	EGFR-TKIs	EGFRm ade.	PD-L1≥50%		16					1.6	10.1	
				PD-L1<1%		86					7.3	38.2	
Yang (41)	2020	EGFR-TKIs	EGFRm ade.	PD-L1≥50%		18		38.9%			5.9		1/10 (10%)
				PD-L1 1-49%		39		56.4%			12.8		5/14 (35.7% )
				PD-L1 0%		96		65.6%			12.5		36/67 (53.7%)
Brown (42)	2019	Osimertinib (First)	EGFRm NSCLC	PD-L1 positive		28				79%	18.4		
				PD-L1 negative		26				85%	18.9		
		Erlotinib/gefitinib (First)	EGFRm NSCLC	PD-L1 positive		24				71%	6.9		
				PD-L1 negative		28				82%	10.9		
BIM													
Takeuchi (43)	2019	Gefitinib + Vorinostat (≥ Second)	EGFRm NSCLC	Concurrent with BIM deletion polymorphism		12				83.3%	5.2		

N, number; ORR, objective response rate; DCR, disease control rate; PFS, progression-free survival; mo, months; OS: overall survival; TKIs, tyrosine kinase inhibitors; EGFRm, EGFR-mutant; ade, adenocarcinoma; NSCLC, non-small cell lung cancer; NR: not reached; VAF: variant allelic frequency.

## Common Concurrent Genetic Alterations

### Complex EGFR Mutations (Intra-EGFR Co-Mutations)

In-frame deletion mutations in exon 19 (19del) and the Leu858Arg (L858R) point mutation in exon 21 are two major types of EGFR activating mutations with an ORR of 70–80% and PFS of approximately 9–20 months for EGFR-TKIs. These mutations are sensitive or classical (*i.e.*, typical or common) EGFR mutations (4–6, 44). Occasionally, complex mutations occur, meaning a single tumor sample has two or more different EGFR mutations (45–50). The frequency of complex mutations is 3–7% (25, 26, 50–54). Theoretically, the introduction of an additional mutation could change the molecular conformation of the EGFR tyrosine kinase domain, leading to increased or decreased TKI affinity that subsequently affects the clinical outcome (55). Zhang et al. (25) identified 187 patients with complex EGFR mutations out of 5898 EGFR-mutant NSCLC patients. Fifty-one of these patients had advanced lung adenocarcinoma and were treated with first-generation EGFR-TKIs as first-line therapy. The median PFS was 9.5 months. A total of 46 of the patients were evaluated for response. The ORR was 52.2%, and the disease control rate (DCR) was 71.7%. These patients were further divided into four groups: group A (n = 12), patients with double classic mutations; group B (n = 15), patients with classical mutations plus an atypical mutation; group C (n = 7), patients with double atypical mutations; group D (n = 12), patients with complex mutations harboring a primary T790M mutation or an exon 20 insertion. The ORRs for these four groups were 75, 60, 71, and 8.3%, respectively. The PFS values were 18.2, 9.7, 9.6, and 1.4 months, respectively. According to these results, patients with double classic mutations (group A) had the best ORR and PFS. Based on a literature review, the authors pooled 22 patients with concurrent 19del and L858R mutations treated with TKIs. Their ORR and PFS were 80% and 13.9 months, respectively. Consistent with other studies, they observed that patients harboring complex mutations with a classic mutation had similar clinical outcomes to those with single classical mutations (26, 27). In contrast, lower TKI efficacy was observed in patients with uncommon complex mutations, especially those with a primary T790M mutation or exon 20 insertions (27).

As the Zhang et al. (25) study showed, patients harboring complex mutations with a primary T790M or exon 20 insertion predominantly presented with primary EGFR-TKI resistance accompanied by the poorest ORR and PFS. Similar results were observed in other studies, which revealed that first and second-generation EGFR-TKIs were ineffective in patients with primary T790M mutations (PFS <3 months) (56, 57). Osimertinib showed better efficacy, with a PFS and OS of 17.0 months (95% CI, 14.0–20.0 months) and 29.9 months, respectively, for 18 patients with a primary T790M mutation (58). Another study by Zhang et al. (59) demonstrated that for 31 patients harboring compound EGFR mutations with primary T790M or exon 20 insertion and sensitive mutations, the median PFS for the patients treated with osimertinib (n = 15) was longer than that for patients treated with first-generation EGFR-TKIs (n = 16) (18.0 *vs*. 1.2 months; p < 0.001).

Previous studies showed that first-generation EGFR-TKIs had poor efficacy in patients with uncommon mutations (alone or as part of a compound mutation) (60, 61), while second-generation TKI afatinib had better activity (ORR 71.1%; median PFS 10.7 months) (57). Thus, afatinib has been approved by the United States Food and Drug Administration as first-line treatment for patients with uncommon EGFR mutations. A recent pooled analysis confirmed the efficacy of first-line afatinib in patients with uncommon compound mutations (n = 35), with an ORR of 77.1% and a median DOR of 16.6 months (62). In addition, osimertinib demonstrated moderate efficacy with an ORR of 50% and a median PFS of 8.2 months (62). Furthermore, some studies found that these patients had a significantly lower incidence of an acquired T790M mutation (63). Considering that patients with complex EGFR mutations and a secondary T790M mutation have a shorter PFS and OS when subsequently treated with osimertinib (64), first-line osimertinib is a choice for these patients. Additional studies on EGFR-TKIs efficacy focused specifically on this subgroup of patients are needed.

Collectively, it is difficult to analyze the efficacy of EGFR-TKIs in patients with uncommon complex EGFR mutations because almost 200 EGFR mutation types have been identified so far, and uncommon mutations have great heterogeneity (25, 65). It appears that afatinib and osimertinib are better options for these patients; however, more studies with larger populations are needed. Osimertinib provided a great survival benefit for patients harboring complex mutations with a primary T790M or an exon 20 insertion that had *de novo* resistance to first and second-generation TKIs and should be taken into consideration for treatment for this subset of patients.

### TP53 Mutations

p53 can induce cell cycle arrest, senescence, and apoptosis and, thus, regulates the response to various cellular stress signals (66). Mutations in the tumor suppressor TP53 gene, which encodes p53, are found in 35–55% of NSCLC cases, more prevalent in squamous cell carcinoma than adenocarcinoma (67), and highly correlated with smoking habits (68). TP53 mutations may have a negative prognostic effect for NSCLC (69).

TP53 is the most prevalent co-alteration observed in EGFR-mutant NSCLC patients, with a frequency of 55–65% (8, 28, 70). Preclinical studies have shown a link between TP53 status and EGFR-TKI response (71–73). Apoptosis induced by gefitinib in NSCLC cell lines requires wild-type p53, which can induce Fas and caspase-dependent cell death, thus increasing TKI sensitivity. In contrast, gefitinib-induced apoptosis is reduced in mutated p53 cells (71). Some *in vitro* models of other tumor types also demonstrated a correlation between TKI response and TP53 mutation, especially in urothelial carcinoma (74, 75). Several studies revealed that patients with coexisting TP53 mutations treated with EGFR-TKIs showed a trend toward lower ORR, shorter PFS, and OS compared to patients with wild-type TP53 that did not reach statistical significance (28–31, 68). However, a recent meta-analysis indicated significantly poorer prognosis for patients with concurrent TP53 mutations treated with first-line EGFR-TKIs (pooled HRs for PFS and OS of 1.69 and 1.94, respectively) (76).

Interestingly, a study focused on TP53 exon 8 mutations demonstrated reduced responsiveness to EGFR-TKIs and worse prognosis, mainly in patients harboring EGFR 19del (29). The data showed that TP53 exon 8 mutations were associated with a significantly lower DCR (42 *vs*. 87%; p < 0.001) and a trend towards shorter PFS and OS compared to TP53 exon 8 wild-type patients in the whole cohort. These differences in PFS and OS became significant in the EGFR 19del subgroup (median PFS, 4.2 *vs*. 16.8 months; p < 0.001; median OS, 7.6 months *vs*. not reached; p = 0.006). In addition, patients with TP53 exon 8 mutations also showed a significantly higher risk of disease progression or death than TP53 exon 8 wild-type patients in this subgroup (HR for PFS, 6.99; 95% CI, 2.34–20.87; p = 0.006; HR for OS, 4.75; 95% CI, 1.38–16.29; p = 0.013).

Labbé et al. (31) found that the PFS for patients with TP53 missense mutations was significantly shorter than for TP53 wild-type patients. This study demonstrated that the patients with concurrent EGFR and TP53 mutations who progressed on EGFR-TKIs treatment (n = 24) were less likely to have a secondary EGFR T790M mutation [45% (5/11) *vs*. 69% (9/13); p = 0.41]. These data are similar to the recent work of VanderLaan et al. (30) who also showed a trend towards a decreased acquired T790M rate in tumors with concurrent TP53 mutations. Among the patients with secondary T790M, 12 received third-generation EGFR-TKIs (11 were evaluable). The ORR was not significantly different between TP53 mutant and wild-type patients in this subset [100% (3/3) *vs*. 88% (7/8)] (31). The influence of co-occurring TP53 mutations on the efficacy of third-generation EGFR-TKIs in patients with acquired T790M mutations following initial EGFR-TKI failure has also been explored (28). Surprisingly, patients with TP53 mutations had a significantly shorter PFS and worse OS compared to patients with wild-type TP53 (median PFS, 8.9 *vs*. 12.8 months; p = 0.029; median OS, 17.8 *vs*. 26.6 months; p = 0.007) in this cohort.

Coexisting of certain mutation sites (*e.g.*, exon 8 mutations) or certain types of TP53 mutations (*e.g.*, missense mutations) may be related to poor EGFR-TKI efficacy, and additional studies focusing on these different types of TP53 mutations are needed to confirm their prognostic impact. Nevertheless, it seems that EGFR-TKIs have lower efficacy in patients with concurrent TP53 mutations. A combination of EGFR-TKI with antiangiogenic therapy showed encouraging efficacy for these patients. In the RELAY study, ramucirumab plus erlotinib first-line therapy showed a superior PFS benefit in patients with EGFR-mutant metastatic NSCLC compared to erlotinib alone (19.4 *vs.* 12.4 months; HR, 0.591; p < 0.0001). Thus, this combination may be a better choice for these patients. Indeed, significant PFS improvement was observed in EGFR-mutant (19del/L858R) patients with concurrent TP53 mutations (77). In the ALTER-L004 study (NCT03736837), anlotinib plus icotinib showed encouraging efficacy and good tolerability for previously untreated, EGFR-mutant advanced NSCLC patients. Fourteen patients with TP53 mutations had an ORR of 78.5% and DCR of 100% (78). Moreover, in the ACTIVE phase III study, apatinib plus gefitinib demonstrated superior PFS as first-line therapy in patients with EGFR-activating mutations, and patients with TP53 exon 8 mutations significantly benefited from this combination (HR 0.24) (79).

### PIK3CA Mutation

The PI3K/AKT pathway is a downstream signaling pathway of the HER family that is important in oncogenesis and lung cancer progression (80, 81). PIK3CA encodes the catalytic subunit, and PIK3CA mutations can activate the PI3K/AKT pathway (82). PIK3CA mutations are found in about 2–5% of NSCLC cases (83–85) and are considered rare oncogenic drivers in NSCLC. The majority of mutations occur in exon 9 (E545K, E545Q, E545G, E545A, Q546R, E542K, and T536I) and exon 20 (H1047R, H1047L, M1043L, G1007R, and Y1021C), with E545K and H1047R being the most frequent mutations (32, 33, 86, 87). In contrast to the mutual exclusivity of many oncogenic drivers in lung cancers, PIK3CA mutations frequently coexist with other oncogenic driver mutations, especially EGFR and KRAS (83, 88–90). Indeed, PI3KCA mutations have been found in approximately 3.5% of EGFR mutation-positive patients (91) and appear to be an indicator of resistance and poor survival for NSCLC patients treated with EGFR-TKIs (92). In a preclinical study, the introduction of an activated PIK3CA p.E545K mutation in exon 9 into the EGFR mutation-positive (19del) HCC827 cell line conferred resistance to gefitinib (93).

The role of PIK3CA mutations in predicting the efficacy of EGFR-TKIs was first investigated by Ludovini et al. (92, 94), who reported that six patients with a PIK3CA mutation had a shorter time to progression (TTP, median, 2.3 *vs*. 6.0 months; p = 0.01) and OS (median, 9.9 *vs.* 30.2 months; p < 0.001) after treatment with gefitinib or erlotinib. It should be mentioned that this study included patients with an unselected EGFR mutation status, with 75.3% (125/166) having a wild-type EGFR. Only two of the six PIK3CA-mutant patients had a concurrent EGFR mutation. One patient had an EGFR S784F mutation in exon 20 and experienced progressive disease (PD) on EGFR-TKI treatment. The second patient with EGFR 19del showed a partial response (PR) to erlotinib but experienced treatment failure after four months of therapy.

Three studies directly compared the efficacy of EGFR-TKIs in EGFR-mutant patients with or without concomitant PIK3CA mutations. Eng et al. (32) found that patients with concurrent PIK3CA mutations had a lower ORR (62% vs. 83%; p = 0.80) and shorter median TTP (7.8 *vs*. 11.1 months; p = 0.84) to EGFR-TKIs. However, these differences were not statistically significant, and these two groups had the same median duration of EGFR-TKI therapy (14.6 months; p = 0.65). A prospective study also explored the impact of PIK3CA mutations on the clinical characteristics and treatment response to EGFR-TKIs in lung adenocarcinoma (33). Of the 344 patients enrolled, six had coexisting PIK3CA mutations. These patients had a similar response to EGFR-TKIs as patients with wild-type PIK3CA (ORR, 66.7 *vs*. 78.7%; p = 0.476). Interestingly, these PIK3CA-mutant patients showed a tendency of longer PFS (median, 12.0 *vs*. 8.8 months) and OS (median, 25.1 *vs*. 21.4 months) compared to those with wild-type PIK3CA, although the differences were not significant (p = 0.401 and p = 0.247, respectively). These data are contrary to the Eng study described above (32). However, a recent study analyzing eight patients with coexisting PIK3CA mutations in a cohort of 54 EGFR-mutant advanced NSCLC patients treated with first-generation EGFR-TKIs found that concurrent PIK3CA mutations were significantly associated with a longer PFS compared to wild-type PIK3CA (95). Interestingly, further study revealed a domain-dependent effect of the PIK3CA mutations on PFS. Mutations in the p85 binding domain (R88Q, R108H, and K111E) were associated with an improved PFS, while mutations in the kinase (Y1021H and H1047R), helical (E542K), and C2 (N345K) domains were associated with a worse PFS (95). Although these findings need to be validated in larger cohorts, they provide a clue for understanding the controversial results of the described studies.

In summary, the available PIK3CA studies had limited sample sizes and inconsistent results. Therefore, additional studies on PIK3CA mutations in different domains and their impact on EGFR-TKI efficacy in a larger population are needed. Based on the present results, we believe that decision-making regarding whether to initiate EGFR-TKI therapy in the clinic should not be affected by the presence of a concurrent PIK3CA mutation.

### PTEN Alterations

Gene of phosphate and tension homolog deleted on chromosome ten (PTEN) is a tumor suppressor gene and master negative regulator of the PI3K/AKT pathway (81, 96). PTEN inactivation, which can be caused by several mechanisms (*e.g.*, decreased protein levels, mutations, loss of heterozygosity, and epigenetic silencing (97)), plays an important role in lung cancer oncogenesis and progression (81, 96). Indeed, it is a frequent event in NSCLC. Such a loss in PTEN function can constitutively stimulate the PI3K/AKT pathway and increase cellular proliferation (96) and may be associated with EGFR-TKI sensitivity (98–100).

Loss of PTEN occurs in more than 40% of NSCLC cases and is associated with poor clinical outcome (101–104). This association appears to be true for EGFR-mutant patients treated with EGFR-TKIs. Wang et al. (105) discovered that for 169 advanced NSCLC patients who harbored EGFR-sensitive mutations treated with EGFR-TKIs, patients with concurrent PTEN deletion had a shorter PFS and OS than those with intact PTEN (HR for PFS, 3.64; 95% CI, 1.47–9.00; HR for OS, 2.86; 95% CI, 1.04–7.89). In addition, both PTEN deletion (HR, 4.29; 95% CI, 1.72–10.70) and low PTEN protein expression (HR, 1.96; 95% CI, 1.22–3.13) were independent predictors of worse PFS for patients treated with EGFR-TKIs. In contrast, high PTEN expression has been reported to be a significant favorable prognostic marker (104). Endoh et al. (106) studied 78 patients with recurrent disease after surgical resection who were treated with gefitinib, and found that high PIK3CA and PTEN expression levels were associated with prolonged OS. In addition, the longest OS was observed in EGFR-mutant patients with concomitant high PTEN expression.

PTEN mutation is rare in NSCLC (2–5% of adenocarcinomas) but also a poor prognostic factor for EGFR-TKI treatment. Kim et al. (28) reported three patients with concurrent PTEN mutations out of 82 patients who acquired the T790M mutation following initial EGFR-TKI failure. They observed that the PTEN mutation was associated with significantly shorter PFS (median, 2.6 *vs*. 10.3 months; p = 0.001) and worse OS (median, 13.2 *vs.* 24.3 months; p = 0.005) for third-generation EGFR-TKIs.

These results provide some information about the efficacy of EGFR-TKIs in patients with concurrent PTEN alterations; however, further research is needed to confirm these findings to determine whether they can assist clinical selection for EGFR-TKI treatment.

## Concurrent Driver Gene Alterations

### ALK Rearrangement

The anaplastic lymphoma kinase (ALK) rearrangement is an oncogenic driver that occurs in approximately 5% of NSCLC patients (107). ALK-TKIs are recommended as first-line therapy for these patients. Early studies have suggested that ALK rearrangements are mutually exclusive with EGFR mutations (108). However, recent reports have described the incidence of concomitant EGFR mutations and ALK rearrangement at a rate of 0.45–1.6% in patients with NSCLC, accounting for 3.9–13.6% of EGFR-mutant and 15.4–18.8% of ALK-rearranged patients (17, 34, 36, 109). With the increased use of next-generation sequencing (NGS)-based mutational profiling, the detection of co-alterations is expected to increase.

EGFR-TKIs and ALK-TKIs play irreplaceable roles in treating NSCLC patients with single oncogenic driver alterations; however, their effects are controversial in double-positive patients. Yang et al. (34) revealed that 13 out of 977 NSCLC patients had co-altered EGFR and ALK. Ten patients received first-line EGFR-TKIs, with an ORR of 80% and a median PFS of 11.2 months (95% CI, 5.6–16.8). Four patients received crizotinib, with three of them first receiving first-line EGFR-TKIs. Of these four patients, two responded to EGFR-TKI but not to crizotinib, while one had *de novo* resistance to the EGFR-TKIs but was responsive to crizotinib. The fourth patient, who received first-line crizotinib, achieved a PR and 15.1 months of PFS but did not respond to subsequent EGFR-TKI treatment. The authors further found that the diverse responses to the ALK- and EGFR-TKIs observed in the ALK/EGFR co-altered patients were associated with phospho-EGFR and phospho-ALK levels. Overall, it seems that patients with EGFR/ALK co-alterations had more favorable responses to first-line EGFR-TKIs in this study.

Lou et al. (35) showed that the ORR (p = 0.57) and median PFS (HR, 0.95; 95% CI, 0.49–1.84; p = 0.87) were 80% and 11.2 months for EGFR/ALK-co-altered patients (n = 10) treated with first-line EGFR-TKIs and 65.5% and 13.2 months for single EGFR-mutant patients (n = 84). A less favorable result for second or further-line crizotinib therapy was found for double-positive patients (n = 5), with an ORR (p = 0.29) of 40% and a median PFS of 1.9 months (HR, 0.40; 95% CI, 0.15–1.10; p = 0.08) compared to 73.9% and 6.9 months for single ALK-rearranged patients (n = 23). The median OS for the single EGFR-mutant, single ALK-rearranged, and EGFR/ALK co-altered patients were 21.3, 23.7, and 18.5 months, respectively (p = 0.06). There was a statistically significant difference in OS between the single ALK-rearranged and EGFR/ALK co-altered patients (p = 0.03). Five EGFR/ALK co-altered patients received sequential treatment with both EGFR-TKIs and crizotinib. Of the four patients that received EGFR-TKI treatment followed by crizotinib, three had good responses and prolonged survival with the first-line EGFR-TKIs but had primary resistance to subsequent crizotinib. The patient that received crizotinib followed by EGFR-TKIs benefited from the crizotinib treatment, but not from the subsequent EGFR-TKI treatment. They also found that after developing resistance to the EGFR-TKIs, activation of ALK may be lower than the EGFR signaling pathway and the abundance of ALK rearrangement may be lower, which could account for the lack of crizotinib efficacy in EGFR/ALK co-altered patients. According to these two studies, first-line EGFR-TKI seems to be a reasonable therapy for EGFR/ALK co-altered patients and single ALK-TKI might be excluded after progressing on EGFR-TKIs. However, these studies contained a limited number of EGFR/ALK co-altered patients who used ALK-TKI as first-line therapy.

A recent study by Zhao et al. (36) presented conflicting results. They identified 26 cases (0.45%) of concomitant EGFR mutations and ALK rearrangement among 5816 NSCLC patients. There were no statistically significant differences in the ORR and PFS for EGFR-TKI treatment between patients with EGFR mutations alone and EGFR/ALK double-positive patients (ORR, 62.1% [59/95] *vs.* 63.2% [12/19]; p = 0.93; median PFS, 11.4 *vs.* 10.3 months; p = 0.87). Additionally, the ORR and median PFS for crizotinib were 65% (39/60) and 12.5 months for ALK-rearranged alone and 66.7% (8/12) and 11.1 months for double-positive patients, respectively. No statistically significant differences were found between these two groups (ORR, p = 1.00; HR for PFS, 1.39; 95% CI, 0.69–2.80; p = 0.28). Furthermore, nine patients were treated with both EGFR-TKIs and crizotinib, eight patients received crizotinib after progression on EGFR-TKI treatment, and the remaining patient received crizotinib before the first-generation EGFR-TKIs. In this subgroup, the ORR was 55.6% (5/9) for EGFR-TKIs and 66.7% (6/9) for crizotinib. A median PFS of 15.0 months was observed when crizotinib was used as a sequential therapy after failure with EGFR-TKI. Crizotinib was administered as first-line therapy in four patients (three received crizotinib monotherapy, and one received crizotinib combined with first-generation EGFR-TKI). The ORR in this subgroup was 75.0% (3/4). These results demonstrated that first-generation EGFR-TKIs and crizotinib in patients with concomitant EGFR and ALK alterations were as efficacious as in patients with single driver gene alterations. Furthermore, contrary to the first two studies mentioned above, crizotinib efficacy as a subsequent therapy after EGFR-TKI treatment failure was not influenced by the EGFR-TKIs. Thus, sequential treatment with EGFR-TKIs and crizotinib could be considered in EGFR and ALK double-positive patients. However, a study in Chinese patients found that EML4-ALK/EGFR and non-EML4-ALK/EGFR co-alterations displayed different clinical characteristics and responses to EGFR-TKIs. Non-EML4-ALK co-alterations are likely to occur as a resistance mechanism against the EGFR-TKIs, and dual-TKI therapy instead of single-TKI therapy might be a better choice for this subset of patients (110).

In summary, EGFR/ALK co-alterations can define a specific subgroup with tumor heterogeneity and diverse responses. Given the limited number of patients in these studies, it was not feasible to determine the best treatment strategy. There are different theories about the coexistence of EGFR mutations and ALK rearrangement in NSCLC—the two gene alterations coexist in different areas (*i.e.*, different cells) of the tumor (111) or are present in the same tumor cells (112). These two scenarios can be distinguished by fluorescence *in situ* hybridization (FISH) and immunohistochemistry (IHC). If the two alterations exist in different tumor cells, there might be a dominant driver clone. Thus, which agent becomes more effective might depend on the levels of the relevant gene alterations. Detection of the abundance of EGFR mutations and ALK rearrangements, levels of phosphorylation of EGFR and ALK and downstream proteins, their dynamic changes, and re-biopsy after progression might help guide the treatment selection and predict the efficacy of TKIs in clinical practice (34, 35). If tumor cells carry both EGFR and ALK alterations, a combination of both TKIs may be a potentially reasonable choice. Still, it is a challenging issue and requires detailed investigation.

### KRAS Mutation

KRAS is one of the commonly detected gene mutations in NSCLC and present in approximately 20–30% of lung adenocarcinomas (113, 114). It rarely overlaps with other driver mutations and is thought to cause inherent resistance to EGFR-TKIs (114–117). However, studies have reported that 5.8–35.8% of EGFR-mutant patients have concurrent KRAS alterations (17, 118). Studies on the efficacy of EGFR-TKIs in these double-positive patients have yielded conflicting results.

Zhuang et al. (21) observed an ORR to EGFR-TKIs of 62.5% (5/8) for patients harboring EGFR/KRAS co-alterations and receiving first-line EGFR-TKI therapy. There were no significant differences in the PFS among patients harboring an EGFR/KRAS co-alteration and those harboring a single EGFR mutation (median, 8.2 *vs.* 9.6 months; p = 0.392). This result suggested that KRAS mutations do not influence the efficacy of EGFR-TKI therapy. However, Benesova et al. (20) identified three patients with concurrent EGFR and KRAS mutations. They all had an initial positive response to EGFR-TKI treatment; however, the efficacy did not last long, resulting in PFS of 3, 5, and 7 months. Rachiglio et al. (37) compared the response to EGFR-TKI treatment between 14 patients with concurrent EGFR and KRAS mutations, in which eight cases with dominant variant allelic frequency (VAF) of EGFR mutations relative to KRAS mutations. The patients with a dominant VAF of EGFR mutations had significantly improved PFS (11.09 *vs*. 2.42 months; p = 0.0081) and ORR (57.1 *vs*. 16.7%) compared to the remaining patients with a dominant VAF of KRAS mutations relative to EGFR mutations.

The limited number of patients harboring double EGFR and KRAS mutations does not allow us to draw definitive conclusions. It can be speculated that KRAS and EGFR mutations may be carried by two different clones, with the initial response due to the elimination of the TKI-sensitive clones by the targeted therapy. However, the growth of the remaining KRAS-resistant clones may lead to a shorter PFS (16). Overall, these studies suggested that EGFR-TKIs can be considered as therapy for treating patients with EGFR/KRAS co-mutations. Quantitative assessment of the allelic frequencies of both the EGFR and KRAS mutations might better identify patients who would benefit from EGFR-TKI treatment.

### ROS1 Rearrangements

Oncogenic ROS1 is a well-recognized and targetable driver in NSCLC. ROS1 rearrangements occur in 1–2% of NSCLC patients (119, 120). The triple ALK, ROS1, and MET-TKI crizotinib is highly effective in ROS1-positive NSCLC patients (121, 122). Patients with ROS1 rearrangements concomitantly with EGFR are extremely rare, with a frequency of less than 1% (123). It would be almost impossible to conduct a clinical trial to compare the different therapeutic strategies in this subtype, although isolated cases can provide some information.

Lambros et al. (124) identified ten patients with ROS1 rearrangements and concomitant EGFR mutations that were treated with first-line EGFR-TKIs. Their responses included six PR, two stable diseases (SDs), and two PDs. Second-line crizotinib therapy was used in four patients with progressive disease during EGFR-TKI therapy, with two PR, one SD, and one PD observed. Interestingly, in the Zhuang study (21), two patients harbored triple EGFR/ROS1/KRAS co-alterations. One patient had PD after receiving second-line crizotinib and PR after third-line icotinib (PFS: 27.5 months), whereas the other patient had a PR after receiving gefitinib as first-line treatment (PFS: 12.7 months).

Despite the limited data, recommending EGFR-TKI as the first-line therapy for patients with dual EGFR and ROS1 alterations is reasonable, while crizotinib may be more useful as a second-line treatment after EGFR-TKI progression.

### MET Alteration

Mesenchymal-epithelial transition (MET) receptor is a transmembrane tyrosine kinase. It can activate downstream signaling pathways (*e.g.*, RAS/RAF/MAPK and PI3K/AKT/mTOR) by binding to the ligand hepatocyte growth factor. These pathways play important roles in cell proliferation, survival, migration, motility, and invasion (125–128). MET gene amplification has been recognized as a common mechanism of acquired resistance to EGFR-TKIs (129, 130), which encouraged researchers to pay more attention to the role of MET in the intrinsic resistance to these agents. *In vitro* studies have shown that MET amplification in HCC827 lung adenocarcinoma cells, which harbor EGFR 19del, mediates resistance to EGFR-TKIs (131). In addition, the coexistence of positive MET FISH status and EGFR mutations is associated with shorter DFS and OS after surgery in patients with lung adenocarcinoma (132). However, the relationship between MET FISH status and clinical outcomes for EGFR-TKI treatment is unclear.

Two distinct processes (*i.e.*, polysomy and amplification) lead to MET copy-number gains (133). FISH, used to identify MET status in many clinical trials, can distinguish polysomy and true amplification. True MET amplification causes copy number increases without an increase in centromeric region of chromosome 7 (CEP7). Thus, the MET/CEP7 ratio increases. In polysomy, a MET copy increase is associated with an increase in the corresponding centromere. Therefore, polysomy has a preserved MET/CEP7 ratio (133). No standard criteria for MET positivity have been established. Two commonly used scoring systems are PathVysion (MET/CEP7 ratio ≥ 2), which only includes amplification, and the Cappuzzo scoring system (≥5 MET signals per cell), which includes both polysomy and amplification (132). MET amplification can be detected by FISH, IHC, and NGS. Lack of platform harmonization and thresholds for defining MET positivity has contributed to the conflicting results.

Noro et al. (38) explored the correlations between pre-treatment MET FISH status and OS and PFS in patients with EGFR-mutant lung adenocarcinoma treated with gefitinib. Eleven patients with MET FISH positivity (ten patients exhibited high polysomy [mean MET per cell ≥5 copies]; one patient exhibited amplification [MET gene/CEP7 ≥2 per cell]) had significantly shorter PFS and OS than 24 patients who were MET FISH-negative (PFS, 7.6 *vs*. 15.9 months; p = 0.001 and OS, 16.8 *vs.* 33 months; p = 0.03). Thus, pre-gefitinib MET FISH status may predict shortened PFS and OS.

Lai et al. (22) identified 52 patients with a high MET copy number gain (CNG) using FISH (MET-high: ≥5 copies per nucleus; polysomy: MET/CEP7 <2.0; amplification: MET/CEP7 ≥2.0) in 200 metastatic TKI-naive EGFR-mutant NSCLC patients. A total of 154 patients were treated with first-line EGFR-TKI monotherapy. The ORR was 74.4 and 53.9% in MET-high and MET-low patients, respectively (p = 0.033), while the median time-to-treatment failure (TTF) was similar in these two groups (12.2 *vs.* 13.1 months). However, all five patients with MET amplification had a poor or short-lived response to the EGFR-TKI (median TTF, 5 months; range, 1.0 to 6.4 months), suggesting that MET amplification concurrent with CNG ≥5 affects the response to EGFR-TKI. Furthermore, a patient harboring the EGFR L858R mutation and a coexisting MET amplification with a CNG of 7.3 and MET/CEP7 of 3.4 experienced disease progression within four weeks after starting erlotinib but showed significant regression of a pulmonary lesion when treated with crizotinib monotherapy. Li et al. (23) reported that a patient with coexisting EGFR 19del and *de novo* MET amplification had disease progression after initial treatment with icotinib, but the lung mass shrunk significantly after switching to crizotinib monotherapy. These data suggested that primary MET amplification could be a possible mechanism of intrinsic EGFR-TKI resistance, and patients harboring this genomic alteration may benefit from MET inhibitors.

Preliminary data from several small studies demonstrated crizotinib efficacy in patients with *de novo* MET amplification. In 15 patients with *de novo* MET amplification (A MET/centromere ratio [MET/CEN] ≥1.8), the ORR, PFS, and OS for crizotinib were 73.3%, 6.5 months (95% CI, 2.7–10.3), and 31 months, respectively (134). In addition, MET and EGFR inhibitor combination has had some effect in patients with EGFR-mutant NSCLC that developed resistance to prior EGFR-targeted therapies through MET gene amplification (127). A case report revealed that this combination might also have efficacy in patients with EGFR-mutant NSCLC and concomitant *de novo* MET amplification (24). While the patient had primary resistance to erlotinib, the tumor shrank after the addition of crizotinib to the treatment strategy.

In summary, it is still uncertain whether MET positivity is related to primary TKI resistance in patients with EGFR-mutant NSCLC, mainly due to the limited patient numbers and different evaluating systems. Further study in larger patient populations using uniform criteria to assess MET status is needed. MET inhibitor monotherapy and combinations with EGFR-TKIs appear to be potential treatment strategies for EGFR-mutant patients with primary MET alterations but require further validation.

## Other Biomarkers

### PD-L1 Expression

Programmed death-ligand 1 (PD-L1) is an immune checkpoint protein expressed on tumor and tumor-infiltrating immune cells. PD-L1 expression can be used as a predictive biomarker for PD-1 and PD-L1 blockade therapy (135–138). PD-1/PD-L1 antibodies, including nivolumab, pembrolizumab, and atezolizumab, have been approved for first- or second-line treatment of advanced NSCLC (139). However, PD-1/PD-L1 inhibitors lack efficacy in most NSCLC patients with EGFR mutations (140), even in those with high PD-L1 expression (tumor proportion score [TPS] ≥50%) (141). Positive PD-L1 expression (≥1%) occurs in about 50% of EGFR-mutant tumors, while high PD-L1 expression (≥50%) occurs in about 5%, which is less frequent compared to EGFR mutation-negative tumors (42). In addition, low tumor mutational burden and the lack of CD8+ tumor-infiltrating lymphocytes in the tumor microenvironment in lung cancer with EGFR mutations may be possible explanations for the lack of efficacy of PD-L1 inhibitors (142). A potential relationship may also exist between PD-L1 expression and EGFR-TKI efficacy in EGFR-mutant lung cancer patients.

Preclinical studies have demonstrated that EGFR activation can facilitate immune escape by inducing PD-L1 expression (143). Moreover, PD-L1 expression in EGFR-mutant NSCLC cell lines can be downregulated by EGFR inhibitors (144). A recent study revealed that EGFR-mutant NSCLC cell lines with higher PD-L1 expression were less sensitive to gefitinib. In addition, PD-L1 overexpression may induce epithelial-mesenchymal transition through the activation of the TGF-*β*/Smad canonical signaling pathway, leading to primary resistance to EGFR-TKIs (145).

Earlier clinical studies that examined PD-L1 expression generated conflicting results. Some studies showed that positive PD-L1 expression was significantly correlated with a greater DCR and longer PFS and OS after treatment with EGFR-TKIs, while another study found no significant correlation between PD-L1 expression and efficacy (146–148). Five recent studies in a larger Asian population showed that PD-L1 expression was associated with poor clinical outcomes in EGFR-mutant patients treated with EGFR-TKIs. Both Soo et al. (149) and Yoneshima et al. (150) (n = 90 and 71 EGFR-mutant NSCLC patients, respectively) found that PD-L1 expression was significantly associated with shorter PFS for EGFR-mutant NSCLC patients treated with EGFR-TKIs. Three additional studies revealed that high PD-L1 expression not only predicted a poor response to EGFR-TKIs but was also related to primary resistance to these agents. In a study of 101 patients with EGFR-mutant NSCLC, strong PD-L1 expression (TC3/IC3 ≥50% for tumor cells [TC] or ≥10% for immune cells [IC]) was significantly associated with decreased ORR and shortened PFS compared to weak (TC1-2/IC1-2: 5–49% for TC or 5–9% for IC) or negative (<5% for TC or IC) PD-L1 expression (ORR, 35.7 *vs*. 63.2 *vs.* 67.3%; p = 0.002; PFS, 3.8 *vs*. 6.0 *vs.* 9.5 months; p < 0.001), regardless of EGFR mutation (*e.g.*, 19del or L858R) (39). Furthermore, patients with *de novo* resistance to EGFR-TKIs had a higher proportion of positive PD-L1 expression than those with acquired resistance (66.7 *vs.* 30.2%; p = 0.009).

The study performed by Hsu et al. (40) included 123 EGFR-mutant lung adenocarcinoma patients. The median PFS and OS for the EGFR-TKIs were 1.6 months (95% CI, 1.1–2.0) and 10.1 months (95% CI, 6.4–13.8) in patients with a PD-L1 ≥50%, which were clearly shorter than those for patients with a PD-L1 <1% (median PFS, 7.3 months; 95% CI, 2.7–12.0; median OS, 38.2 months; 95% CI, 26.1–50.3). Therefore, higher PD-L1 expression levels were associated with lower OS and PFS for patients with EGFR mutations treated with EGFR-TKIs. The patients were divided into two groups (primary resistance and disease control). In the primary resistance group, 22.7 and 30.3% of the patients had PD-L1 TPS ≥50 or ≥25%, respectively, while the frequencies were only 1.8 and 3.5%, respectively, in the disease control group (both p < 0.001). These results revealed that a higher PD-L1 expression level was associated with a higher incidence of primary EGFR-TKI resistance.

Yang et al. (41) performed a study with 153 EGFR-mutated lung adenocarcinoma patients. The ORR for EGFR-TKI and PFS was better in patients with PD-L1 expression <50% (ORR/PFS in PD-L1 0 *vs.* 1–49 *vs.* ≥50%: 65.6%/12.5 *vs.* 56.4%/12.8 *vs.* 38.9%/5.9 months, p < 0.05). The multivariate analysis showed that PD-L1 <50% was an independent prognostic factor for longer PFS (HR, 0.433; 95% CI, 0.250–0.751; p = 0.003). Furthermore, a significant proportion of patients with TPS ≥ 50% had primary resistance to EGFR-TKIs (44.4%). In addition, 91 patients were re-biopsied for T790M testing upon disease progression. Tumors with higher PD-L1 expression were less likely to develop an acquired T790M mutation (T790M+ in PD-L1 0 *vs.* 1–49 *vs.* ≥50%: 53.7% (36/67) *vs.* 35.7% (5/14) *vs.* 10% (1/10); p = 0.024).

Interestingly, a recent study by Brown et al. (42) revealed that unlike with erlotinib or gefitinib, the clinical benefit of first-line osimertinib treatment in NSCLC patients with EGFR mutations was unaffected by PD-L1 expression status (PFS: PD-L1-positive (TC ≥1%) *vs.* PD-L1-negative patients (TC <1%): 18.4 *vs.* 18.9 months). In addition, it can be inferred from the Yang et al. study (41) that patients with higher PD-L1 expression have less of a chance to use osimertinib as subsequent therapy because they are less likely to develop acquired T790M mutations, suggesting that first-line osimertinib may be a better choice for these patients.

Because immune checkpoint inhibitor (ICI) monotherapy showed poor efficacy in patients with EGFR mutations, different combinational strategies have been explored. Combination therapy consisting of EGFR-TKI with ICIs or sequential use of EGFR-TKI following ICI therapies are not proper choices because they showed increased grade three or higher toxicities and increased the risk of severe immune-related adverse events (142). Of note, in the IMpower-150 study (n = 124 EGFR-mutant NSCLC patients), atezolizumab plus bevacizumab and chemotherapy (ABCP; n = 34) yielded a higher ORR (71 *vs.* 42%), DOR (11.1 *vs.* 4.7 months), PFS (10.2 *vs.* 6.9 months; HR, 0.61; 95% CI, 0.36–1.03), and OS (not evaluable [NE] *vs.* 18.7 months; HR, 0.61; 95% CI, 0.29–1.28) compared to bevacizumab plus chemotherapy (BCP; n = 45) (151). In addition, toripalimab, a PD-1 inhibitor, in combination with chemotherapy, showed promising anti-tumor activity with a manageable safety profile in a phase II prospective clinical trial (152). These results suggest that chemotherapy and antiangiogenic therapy may be related to EGFR cancer immunity; however, additional studies are required to fully understand this relationship. Several Phase III prospective clinical trials are ongoing to explore the efficacy and safety of the combination of immunotherapy with chemotherapy and/or antiangiogenic therapy in EGFR-mutant NSCLC progressed on EGFR-TKIs, including KEYNOTE-789, KEYNOTE-722, ORIENT-31, and JS001 study (NCT03924050).

### BIM Polymorphism

BIM, also known as B-cell chronic lymphocytic leukemia/lymphoma (Bcl-2)-like 11 (BCL2L11), is a BH3-only pro-apoptotic member of the Bcl-2 family. BIM gene products containing BH3 domains are required for the induction of apoptosis by EGFR-TKIs (153–157). The BIM deletion polymorphism is a 2,903-bp fragment deletion in intron 2 of the BIM gene. It results in splicing of exon 3 over exon 4 in the BIM pre-mRNA, generating an inactive BIM protein isoform lacking the crucial BH3 domain. This BIM deletion polymorphism occurs in 12–16% of lung cancer patients with EGFR mutations (158, 159). It impairs EGFR-TKI-related apoptosis and mediates intrinsic resistance to EGFR-TKIs in EGFR-mutant NSCLC cell lines (154).

The impact of the BIM deletion polymorphism on the clinical outcome of NSCLC patients with EGFR mutations treated with EGFR-TKIs has been evaluated in multiple studies with contradictory results. Some studies have shown that EGFR-mutant NSCLC patients with the BIM deletion polymorphism had inferior EGFR-TKI efficacy compared to those with wild-type BIM (154, 160–164). Others found that EGFR-mutant patients with and without the BIM deletion polymorphism had similar clinical outcomes in response to EGFR-TKI treatment (165–168). Several meta-analyses have indicated that the BIM deletion polymorphism is associated with poor ORR (168–172), consistent with other studies demonstrating that the BIM deletion polymorphism is associated with shorter PFS in patients with NSCLC harboring EGFR mutations who received EGFR-TKIs (168–175). Only one study showed no significant association between BIM status and the response to EGFR-TKIs (173).

These results suggested that BIM deletion polymorphism can be used as a predictive biomarker for EGFR-TKI treatment. Patients with the BIM deletion polymorphism may benefit less from EGFR-TKI therapy. EGFR-TKI combination therapy with histone deacetylase (HDAC) inhibitors may be one approach to overcome the inferior outcomes conferred by the BIM deletion. A recent phase I study with 12 patients treated with the combination of gefitinib and vorinostat (a small-molecule HDAC inhibitor) revealed a DCR of 83.3% (10/12) at six weeks, with a median PFS of 5.2 months (95% CI, 1.4–15.7) (43). In addition, retrospective analysis revealed that EGFR-TKIs plus chemotherapy conferred a significantly higher ORR (65.5 *vs.* 38.9%, p = 0.046), prolonged PFS (7.2 *vs.* 4.7 months; p = 0.008) and a longer OS (18.5 *vs.* 14.2 months; p = 0.107) compared to TKIs alone in advanced NSCLC patients with EGFR mutations and BIM deletion polymorphism (176). The latter difference did not reach statistical significance. Further studies are needed to determine the clinical efficacy of these combined therapies.

## Conclusions

This report described the many studies on the efficacy of EGFR-TKIs in patients with EGFR-mutant NSCLC with coexisting intra-EGFR or other gene mutations and molecular markers that may predict EGFR-TKI efficacy. Most of these studies were retrospective studies with controversial results and inconsistencies between sample size, race, disease stage, and treatment line. In addition, tumor heterogeneity and a combination of several concurrent genes were not considered. Nevertheless, we presented additional information in this field and have provided clues for clinicians to identify which patients might be more effectively treated with EGFR-TKIs, and those for which EGFR-TKIs would be less effective, even though they have sensitive EGFR mutations. We also described some available treatment strategies for patients who might not benefit from EGFR-TKIs. To the best of our knowledge, this is the first review on EGFR-TKI efficacy encompassing so many specific concurrent gene mutations and other biomarkers. To initiate appropriate therapies for each individual patient in the future, the use of comprehensive genomic profiling should become routine. In addition, larger populations with advanced NSCLC stratified by uniform stage and therapy are needed to examine these concurrent alterations and validate their importance as prognostic factors for EGFR-TKI therapy in EGFR-mutant patients.

## Author Contributions

YG wrote the original draft and did the largest amount of revising and editing. JS, YW, LH, LS, JZ, SZ, WJ, and JM collected related literature and did substantial editing of the manuscript. CH oversaw and coordinated the entire process and dramatically altered the orientation of the manuscript. All authors contributed to the article and approved the submitted version.

## Funding

This study was supported by grants from the 345 Talent Project of Shengjing Hospital.

## Conflict of Interest

The authors declare that the research was conducted in the absence of any commercial or financial relationships that could be construed as a potential conflict of interest.
